# Supplementary material for: Epstein-Barr Virus miR-BART1-3p Regulates the miR-17-92 Cluster by Targeting E2F3
Source: Int J Mol Sci. 2021 Oct 10;22(20):10936. doi: 10.3390/ijms222010936 (PMC8539899; doi:10.3390/ijms222010936)
Supplement: Supplementary file 1 [file ijms-22-10936-s001.zip › ijms-1347254-supplementary.pdf]

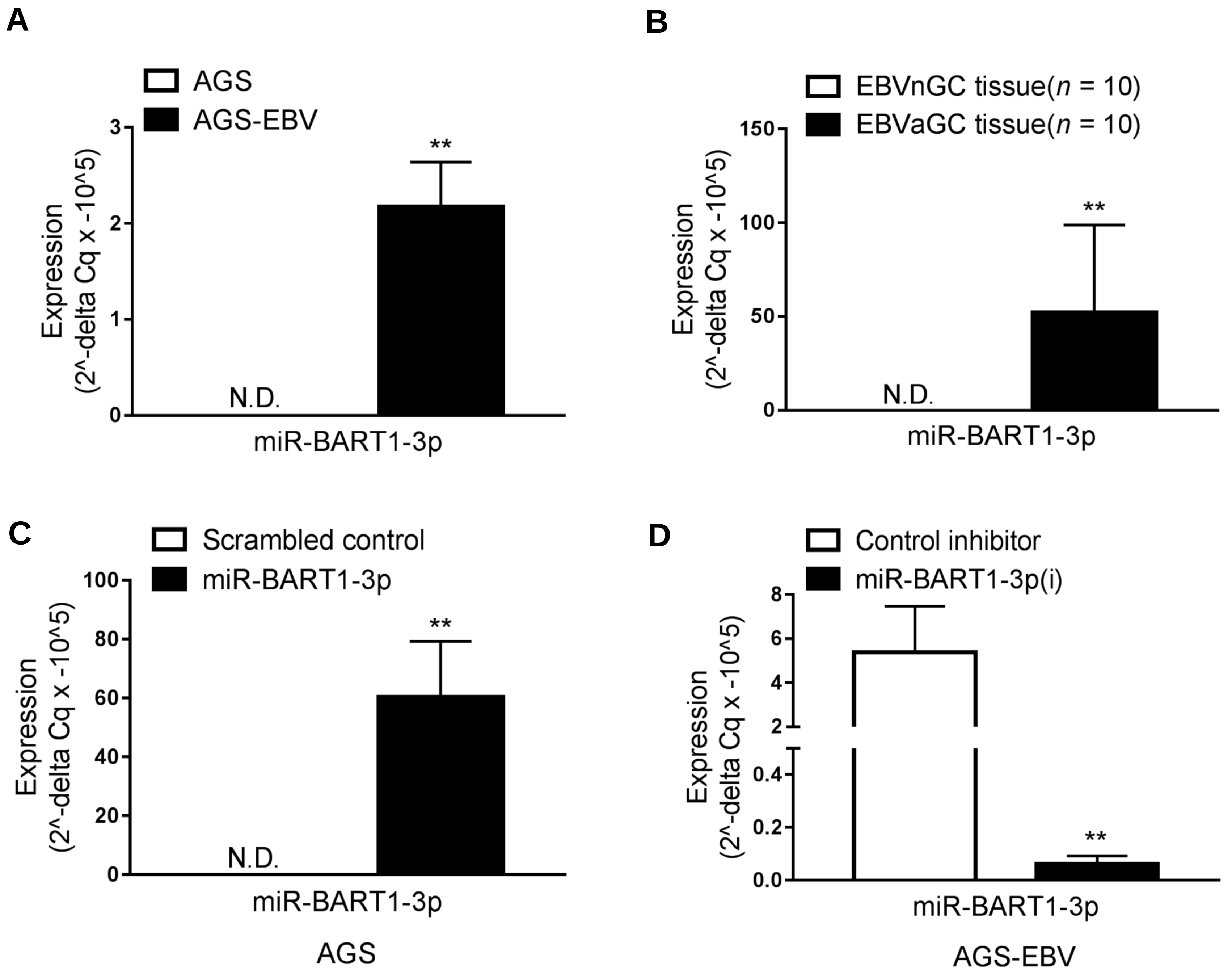

Figure S1. Expression level of miR-BART1-3p. Expression of miR-BART1-3p was assessed by qRT-PCR. Observed Cq values for miR-BART1-3p were normalized using Cq values U6 used as a loading control. When the expression of miR-BART1-3p was undetected, the values were marked as “Not detected (N.D.)” (A) Expression level of endogenous miR-BART1-3p was analyzed in AGS and AGS-EBV cells (n = 3 in triplicate). (B) Expression level of miR-BART1-3p was analyzed using RNA isolated EBVaGC and EBVnGC (n = 10 in triplicate). (C) Expression level of miR-BART1-3p was analyzed in AGS cells following transfection with 30 nM miR-BART1-3p mimic (n = 3 in triplicate). D) Expression level of miR-BART1-3p was analyzed in AGS-EBV cells following transfection with 30 nM miR-BART1-3p(i) (n = 3 in triplicate). Error bars indicate the SD. The data were analyzed using Student's t-test. **, p<0.01.
